# Comparative Analysis of Complication Rates in Tibial Shaft Fractures: Intramedullary Nail vs. Ilizarov External Fixation Method

**DOI:** 10.3390/jcm13072034

**Published:** 2024-03-31

**Authors:** Danilo Jeremic, Nikola Grubor, Zoran Bascarevic, Nemanja Slavkovic, Branislav Krivokapic, Boris Vukomanovic, Kristina Davidovic, Zelimir Jovanovic, Slavko Tomic

**Affiliations:** 1 Institute for Orthopeadics “Banjica”, 11000 Belgrade, Serbia; zoran.bascarevic@iohbb.edu.rs (Z.B.); nemaslav@yahoo.com (N.S.); branislav.krivokapic@iohbb.edu.rs (B.K.); vukomanovicboris@gmail.com (B.V.); drzex@live.com (Z.J.); tomicslavko1956@gmail.com (S.T.); 2 Faculty of Medicine, University of Belgrade, 11000 Belgrade, Serbia; dr.kristina.davidovic@gmail.com; 3 Institute for Medical Statistics and Informatics, Faculty of Medicine, University of Belgrade, 11000 Belgrade, Serbia; nickgrubor@gmail.com; 4 Center for Radiology and Magnetic Resonance Imaging, University Clinical Center of Serbia, 11000 Belgrade, Serbia

**Keywords:** tibial shaft fracture, intramedullary fixation, external fixation, treatment strategies

## Abstract

**Background**: The external fixation (EF) Ilizarov method, shown to offer efficacy and relative safety, has unique biomechanical properties. Intramedullary nail fixation (IMN) is an advantageous alternative, offering biomechanical stability and a minimally invasive procedure. The aim of this study was to assess outcomes in patients undergoing tibia fracture fixation, comparing the Ilizarov EF and IMN methods in an early phase of IMN implementation in Serbia. **Methods:** This was a retrospective study including patients with radiologically confirmed closed and open (Gustilo and Anderson type I) tibial diaphysis fractures treated at the Institute for Orthopedic Surgery “Banjica’’ from January 2013 to June 2017. The following demographic and clinical data were retrieved: age, sex, chronic disease diagnoses, length of hospital stay, surgical wait times, surgery length, type of anesthesia used, fracture, prophylaxis, mechanism of injury, postsurgical complications, time to recovery, and pain reduction. Pain intensity was measured by the Visual Analog Scale (VAS), a self-reported scale ranging from 0 to 100 mm. **Results:** A total of 58 IMN patients were compared to 74 patients who underwent Ilizarov EF. Study groups differed in time to recovery (*p* < 0.001), length of hospitalization (*p* = 0.007), pain intensity at the fracture site (*p* < 0.001), and frequency of general anesthesia in favor of intramedullary fixation (*p* < 0.001). A shorter surgery time (*p* < 0.001) and less antibiotic use (*p* < 0.001) were observed when EF was used. Additionally, we identified that the intramedullary fixation was a significant predictor of pain intensity. **Conclusions:** The IMN method offers faster recovery and reduced pain intensity in comparison to EF, while the length of surgery predicted the occurrence of any complication.

## 1. Introduction

Fractures of the tibial diaphysis represent the most frequently encountered fractures of long bones [1]. With an incidence ranging from 8.1 to 37 per 100,000 individuals annually, these fractures pose a significant public health concern [1]. The vulnerability of these fractures to infection and nonunion is attributed to the absence of sufficient soft tissue coverage and the distinctive vascular supply to the affected region [2]. Furthermore, fractures of the tibial shaft are classified as injuries with severe quality-of-life impact with the potential for enduring disability [2,3].

Conservative management of stable tibial diaphyseal fractures, characterized by closed reduction and cast immobilization, is a common approach [4]. However, this method is not without complications, including an elevated risk of deep vein thrombosis, compartment syndrome, soft tissue damage, and chronic pain due to prolonged immobilization [5]. Despite the lower infection rate associated with conservative cast treatment, it concurrently exhibits the highest incidence of delayed union, nonunion, or inadequate union of fractures [6].

Intramedullary nail fixation is an advantageous alternative, offering biomechanical stability and a minimally invasive procedure [6]. Many experts consider intramedullary nails the treatment for tibial shaft fractures [7,8]. Comparative studies suggest the superiority of intramedullary nail fixation over external fixation in open tibial shaft fractures, mainly when wound closure is promptly executed following nail insertion [8]. The Ilizarov method is also discussed, emphasizing its efficacy and relative safety. This technique’s unique biomechanical properties enable the application of tensioned wires to maintain stable fixation of bone fragments while facilitating fracture site dynamization [9,10,11]. Notable advantages of the Ilizarov method over closed fixation include closed reduction, minimal soft tissue damage, early mobilization, and simplified device removal [12].

The article delves into the controversy surrounding the choice of the most appropriate technique for stabilizing tibial fractures. With its ease of application and minimal impact on blood supply, the utility of external fixation is counterbalanced by a heightened pin tract infection rate, challenges in controlling soft tissue injuries, and a relatively elevated nonunion rate [13]. Conversely, reamed nails offer superior stability but entail a theoretical risk of increased infection and nonunion due to the compromise of endosteal blood supply [2]. Nevertheless, more evidence is required to substantiate this claim since several studies have shown conversely that reamed nails exhibit a higher incidence of union in comparison to non-reamed nails. Notably, the limited number of studies comparing complication rates, including compartment syndrome, poor union, nonunion, and delayed union, in tibial fractures treated with external and intramedullary fixation underscores the need for further research in this domain. Therefore, in this study, we aimed to assess outcomes in patients undergoing tibia fracture fixation, comparing the Ilizarov external fixation method (EF) and intramedullary nail placement (IMN) in an early phase of IMN implementation in Serbia.

## 2. Materials and Methods

This was a retrospective cohort study. Data about consecutive patients with radiologically confirmed closed and open (Gustilo and Anderson type I) tibial diaphysis fracture treated at the Institute for Orthopeadics “Banjica” from January 2013 to June 2017 were collected. The study was approved by the Ethical Committees of the Institute for Orthopedic Surgery “Banjica” (num: 16/2017) and Belgrade University, Faculty of Medicine (num: 2650/IV-16, date: 10 April 2018).

### 2.1. Eligibility Criteria

All consenting consecutive patients aged 18 or older with radiologically confirmed closed and open (Gustilo and Anderson type I) tibial diaphysis fractures, independent of fracture location, were included during the study period. The fractures were radiologically classified using The AO Foundation/Orthopaedic Trauma Association (AO/OTA) fracture classification system. The exclusion criteria were patients who had an open fracture above the Gustilo and Anderson type I classification, who had bone defects, injuries to nerves and blood vessels, and incomplete medical documentation, who were in an alcoholic state on admission, and who had a fracture of the diaphysis of the tibia as part of polytrauma.

### 2.2. Study Design and Data Collection

For patients who underwent external fixation (Ilizarov method) or reamed intramedullary nail fixation, the following demographic and clinical data were retrieved: age, sex, chronic disease diagnoses, length of hospital stay, surgical wait times, surgery length, type of anesthesia used, fracture, prophylaxis, mechanism of injury, postsurgical complications, time to recovery, and pain reduction. Pain intensity was measured by the Visual Analog Scale (VAS), a self-reported scale ranging from 0 to 100 mm [14]. The VAS scale was linguistically adapted to the local cultural area. Pain was evaluated on admission day (prior to surgery) and at last control, i.e., hospital discharge, in accordance with the protocol, at the fracture site and in two nearby joints (knee and ankle).

### 2.3. Surgical Techniques

The surgical choice in this study depended on the preference of the surgeon in charge. The indications for operative technique were the same for both groups, i.e., Gustilo and Anderson type I tibial diaphysis fractures, and surgery was performed independently of open or closed fracture types and fracture location. As IMN was introduced as a novel surgical technique, surgeries were performed by specialized surgeons with at least 5 years of experience performing multiple approaches to tibial diaphysis fractures.

#### 2.3.1. Reamed Intramedullary Nail Fixation

Patients were positioned supine on the operating table to ensure radiolucency and facilitate access for tibial shaft surgery, with the knee flexed at an angle of 90–110°. Anesthesia was administered according to medical indications and the judgment of the anesthesiologist. The surgical area, namely the distal region of the upper leg, lower leg, and foot, was thoroughly sterilized. Prior to surgery, tourniquets were applied to the upper leg of all patients. After disinfecting and garnishing the operative field, we made an incision in the appropriate place on the skin above the tibial fracture (anterior approach to the knee, medial parapatellar approach). After access, we prepared the tibia to receive an intramedullary nail. The key is to precisely guide the intramedullary nail into the tibial canal. We did this under fluoroscopic (X-ray) control to ensure accurate positioning of the nails. After determining the starting point anterior to the articular plateau and medial to the lateral tibial spine and opening the canal, we placed a guide wire. Through the guide wire, we reamed the tibial canal 1.0 mm above the size of the final nail. The surgeon carefully pushed the nail over the guide wire through the tibial canal until it reached the desired position across the fracture. After proper placement of the nail, repositioning of the fracture, and obtaining a satisfactory position, we locked the nail through the guide with two proximal and two distal screws. Once the nail was securely placed and fixed, a final check of the nail’s position was performed via fluoroscopy to ensure that everything was properly positioned. Once the nail was securely placed and fixed, the incision was carefully closed layer by layer. On the first postoperative day, all patients were verticalized with the help of crutches with a support on the operated leg, the wound was bandaged, and the neurovascular status was checked. All patients were included in the early physical rehabilitation program, with walking and support on the operated leg until discharge from the hospital. Regular bandages were applied on the second day.

#### 2.3.2. External Fixation (Ilizarov Method)

Following the administration of anesthesia by the anesthesiologist, the patients were positioned on the operating table in the appropriate supine posture. Buttresses were positioned under the thigh and rear foot to elevate the lower leg to a sufficient height, creating space for the rings to move freely. Following disinfection and preparation of the surgical area (the lower portion of the upper leg and lower leg), we realigned the broken bone and began the process of attaching the Ilizarov external fixator. To achieve accurate positioning of the ring and maximum stability, we used X-ray guidance to attach the rings of the Ilizarov device to the bone using pins. The rings were affixed to the bone with the use of tensioned pins. Once the rings were connected, we inserted a spacer between them to create the outside structure of the Ilizarov external fixator. We affixed these spacers to the rings using screws or other fastening instruments. Once the device was positioned, a final assessment of the realignment of the fracture was conducted using X-rays. The construction of the devices was then carried out, and dressing was applied around the incisions where the pins penetrate the skin. On the first day after surgery, all patients were assisted in standing upright with assistance on the limb that was operated on. The neurovascular condition was assessed, and the wounds around the pins were dressed with bandages. As part of the early physical rehabilitation program, patients engaged in daily activities such as walking and receiving assistance on the leg that had surgery until discharge from the hospital.

### 2.4. Statistical Analysis

Measures of central tendency (mean and median) and variability (standard deviation and percentiles) were used to describe the study population. Categorical features were reported using absolute and relative frequencies. Data analysis used the null hypothesis significance testing paradigm, considering all *p*-values < 0.05 significant. We compared groups using the Student’s *t*-test, Mann–Whitney U test, chi-square test, and generalized linear models. All statistical procedures used SPSS (SPSS for Windows, release 26.0, SPSS, Chicago, IL, USA).

## 3. Results

One hundred thirty-two patients with tibial diaphysis fractures were enrolled and followed in the study. Of the 132 patients, 62.9% were male, while 37.1% were female. The average age was 46.1 ± 16.4 years (range 18–73 years). In our cohort, 56.1% of patients underwent external fixation (Ilizarov method), while 43.9% underwent intramedullary nail fixation. Our samples were balanced concerning sociodemographic characteristics. The characteristics of the cohort are shown in Table 1.

Table 2 shows the types of fractures in total, as well as according to the type of intervention. There were 13 open Gustilo I fractures in the Ilizarov cohort (17.6%) and 9 open Gustilo I in the IMN cohort (15.5%). The 42-A type of fracture was the most common in our study sample (71.2%), followed by 42-B (19.0%). Comminuted fractures were more present in the EF group (20.3%); however, no statistical significance was found in the type of fracture according to the type of intervention (*p* = 0.079).

Patients who underwent external fixation (Ilizarov method) had significantly longer hospital stays than patients who underwent intramedullary nail fixation, *p* = 0.007. No statistically significant difference was found when comparing groups by surgical wait times, mechanism of injury, or frequencies of different fracture types (*p* > 0.05). Patients who underwent intramedullary nail fixation had a significantly higher rate of procedural general anesthesia than patients who underwent external fixation (Ilizarov method), *p* < 0.001. We found a statistically significant difference in median surgical lengths among the two groups. Patients undergoing intramedullary nail fixation had significantly longer procedure lengths than those undergoing external fixation, *p* < 0.001. The two groups also differed in the rate of antibiotic usage, with those undergoing intramedullary nail fixation having higher frequencies of antibiotic use than those undergoing external fixation (Ilizarov method). These differences and their respective *p*-values are shown in Table 3.

No statistically significant difference in frequencies of postoperative complications was found between the two study groups (*p* > 0.05). A statistically significant difference in recovery time was found favoring the intramedullary nail fixation group compared to the external fixation group (*p* < 0.001).

**Figure 1:**
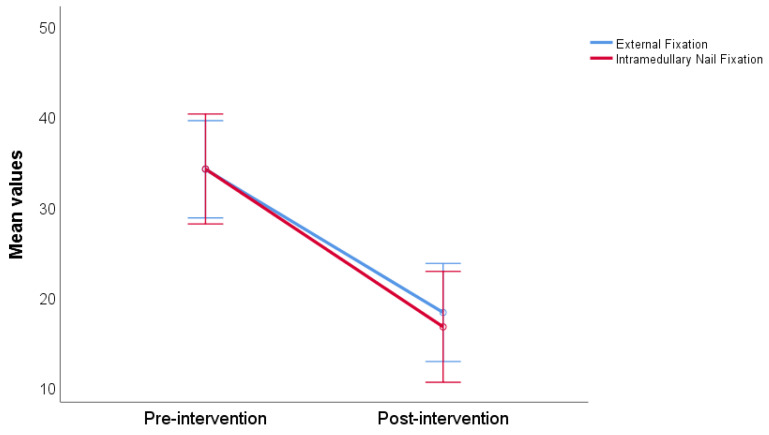
Non-ambulatory knee pain compared by pre- and post-treatment, stratified by intervention type.

The average reported non-ambulatory knee pain before surgical intervention was 34.13 ± 18.45 in the group intended for external fixation (Ilizarov method) and 34.17 ± 18.26 in the group intended for intramedullary nail fixation. The average reported postsurgical non-ambulatory knee pain was 18.26 ± 18.17 and 16.67 ± 18.94 for both groups. Both groups experienced a statistically significant reduction in knee pain after surgery (*p* < 0.001). There was no statistically significant difference between groups in pre- and post-intervention knee pain measurement (*p* = 0.619) (Figure 1).

**Figure 2:**
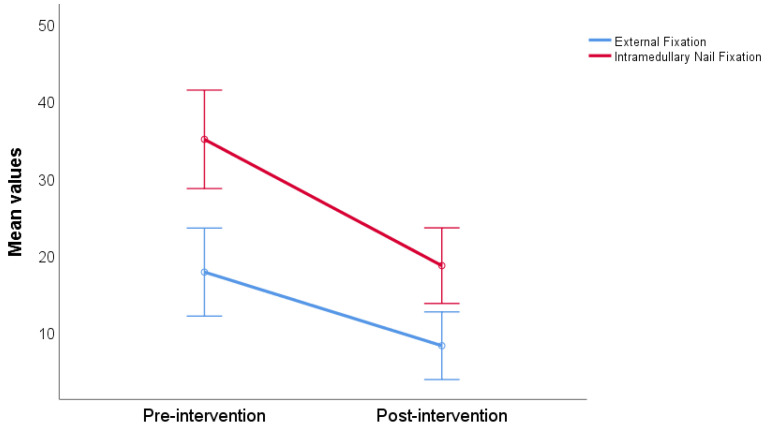
Non-ambulatory ankle pain compared by pre- and post-treatment, stratified by intervention type.

The average reported non-ambulatory ankle pain before surgical intervention was 17.78 ± 19.53 in the group intended for external fixation (Ilizarov method) and 35.0 ± 18.90 in the group intended for intramedullary nail fixation. The average reported postsurgical non-ambulatory ankle pain was 8.22 ± 11.73 and 18.61 ± 17.91 for both groups. Both groups experienced a statistically significant reduction in ankle pain after surgery (*p* < 0.001). There was no statistically significant difference between groups pre- and post-intervention ankle pain measurement (*p* = 0.056) (Figure 2).

**Figure 3:**
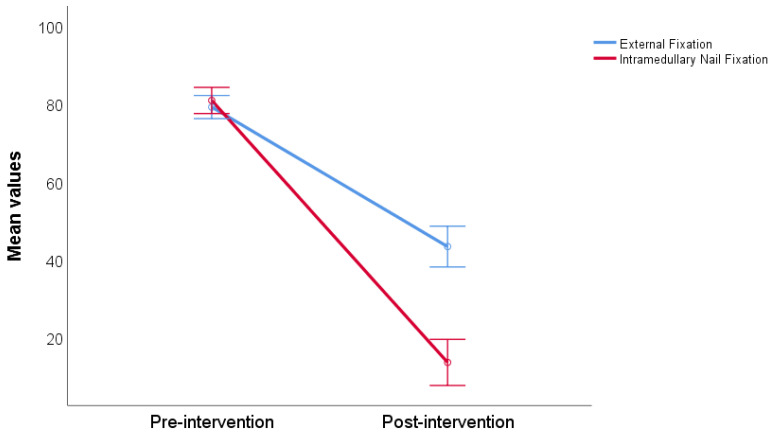
Non-ambulatory fracture site pain compared by pre- and post-treatment, stratified by intervention type.

The average reported non-ambulatory pain at the fracture site before surgical intervention was 79.13 ± 10.92 in the group intended for external fixation (Ilizarov method) and 80.83 ± 9.06 in the group intended for intramedullary nail fixation. The average postsurgical non-ambulatory pain at the fracture site was 43.26 ± 20.88 and 13.47 ± 13.08 for both groups, respectively. Both groups experienced a statistically significant reduction in ankle pain after surgery (*p* < 0.001). Additionally, we found a statistically significant difference between pre- and post-intervention fracture site pain measurement (*p* < 0.001), with the group that underwent intramedullary nail fixation reporting significantly lower fracture site pain scores (Figure 3). There were no other significant predictors found for pain intensity after the surgical intervention in the logistic regression model (*p* > 0.05 for all assessed variables).

We used univariant robust regression to assess variables, with time to recovery as the outcome. We found that intramedullary nail fixation was a significant predictor of recovery (β = −1.08, *p* < 0.001). Table 4 shows the β coefficients with their relevant *p*-values.

We performed univariate screening for predictors of surgical complications with an exact logistic regression model. The statistically significant predictor of any surgical complication was the length of surgery (β = 0.01, *p* = 0.015). Table 5 shows the β coefficients with their relevant *p*-values.

## 4. Discussion

Our study aimed to assess outcomes in patients undergoing open and closed tibia fracture fixation, comparing the Ilizarov EF method and IMN in an early phase of IMN implementation in Serbia. We found no statistically significant difference in rates of postoperative complications between the two groups but noted longer hospitalization and recovery times in the Ilizarov group. Conversely, IMN patients experienced longer surgery durations and a higher frequency of antibiotic use. The study identified (EF) placement as a predictor for pain reduction at the fracture site and faster recovery, while longer surgery duration was associated with more frequent complications.

It remains uncertain whether surgical interventions, such as IMN or EF, result in better outcomes than conservative closed management with casting [15,16,17,18]. A meta-analysis of studies that compared cast treatment versus open reduction and internal fixation or intramedullary nailing of closed tibial shaft fractures found insufficient evidence to support the superiority of any approach [16]. Another review that pooled data from prospective studies of cast versus operative treatment in 895 fractures was also inconclusive [17]. Even if IMN is shown to be better than EF, there exists a lack of consensus regarding the best type of technique for IMN of the tibial shaft in adults [19]. A previous meta-analysis showed that IMN may be superior to other fixation strategies for open tibial shaft fractures. Using unreamed instead of reamed nails may be advantageous in setting open fractures. However, as with previous studies, confidence intervals around pooled malunion and infection risk estimates were extensive, and no recommendation could be given [20].

A recent updated meta-analysis pooling 16 randomized controlled trials found that IMN resulted in a lower rate of postoperative superficial infection and malunion rate but a higher hardware failure occurrence than EF. Additionally, the meta-analysis found no difference in union time, delayed union or nonunion rate, and postoperative deep infection rate between treatments [21]. Our study’s findings agree with this meta-analysis regarding complication rate comparisons. It is still the case, though, that a small number of studies dominate effect sizes regarding certain quality-of-life and functional measures. Evidence synthesis in this field suffers from highly heterogeneous and uncertain data.

In our study, patients who underwent IMN fixation experienced a significantly longer surgery length and a higher frequency of antibiotic use. We identify the duration of surgery as a statistically significant predictor of postsurgical complications, with longer surgeries associated with higher complication rates. Though the effect of surgery length has not been directly compared for tibial shaft fractures, one study found that prolonged operative time increases the infection rate in tibial plateau fractures [22]. The method of fixation used does not seem to impact deep infection rates [23,24,25]. There is evidence that segmental tibial fractures might pose a greater deep infection risk with IMN than EF. However, these differences were minor (3% vs. 2.5%) [26]. Lower rates of superficial infection seem to favor IMN compared to EF [23,25]. Since surgery duration is related to case complexity and some studies indicate that surgical timing does not alter infection rates, other causes, such as expansive tissue injury due to more severe fractures, could be the unexplored etiology of this association [27]. Less antibiotic use found in our EF group might be attributed to hospital protocol recommendations for antibiotic use for all patients after the surgery at the start of this study. Intramedullary nailing was first introduced in Serbia in 2013 at the Institute for Orthopedic Surgery “Banjica”, and the surgeons’ lack of familiarity with a novel method might have resulted in prolonged surgical operations and a cautious approach that overestimated the necessity for antibiotics use. In contrast, surgeons were well experienced with the Ilizarov method, an external fixation technique that has been used in our country for the past 30 years. Thus, patients were given less antibiotics, but the study’s hospitalization duration was significantly longer for patients treated with the Ilizarov external fixator than those treated with intramedullary nailing. This can be explained by the need for wound bandages around Ilizarov’s external fixator, particularly around the pins. In addition, patients were hospitalized longer to facilitate rehabilitation and enable them to regain independent mobility via the assistance of physiotherapists. Due to this well-known concept of a learning curve influencing the outcomes of novel surgical techniques, in this study, we have presented the results of the first five years of IMN implementation in Serbia. This recognition of a measure of expertise might be beneficial to readers when assessing specific surgical techniques and their perioperative outcomes in other countries as well as for other surgical techniques. Some studies, such as the one conducted in Tanzania, showed that intramedullary fixation did not decrease treatment costs despite potentially shorter hospitalization periods [24]. In studies comparing IMN and EF in tibial fractures, recovery time is defined as the time to radiographic union [24,28], full weight-bearing [29], and unprotected weight-bearing. Recovery duration was significantly longer in Ilizarov patients in our study. However, there needs to be more consensus on the definition of recovery, which may have influenced different rates of recovery measurement, as some studies emphasize radiographic and others functional recovery [28]. It has been suggested that a composite measure, functional status combined with weight-bearing, could be used as an objective indicator of recovery. Some studies indicate that the type of fixation (IMN vs. EF) did not significantly differ in radiological healing outcomes after one year. In contrast, studies have reported differences in radiographic union scores and timing of visible fracture healing [24].

We continue to have low and uncertain data on functional outcomes and reoperations [16,19,21,25,30]. Our study observed that the IMN procedure predicted lower pain scores at the fracture site. The recent updated meta-analysis found that the composite pain score (from four RCTs) favored EF instead [10,21,28,29,31]. This apparent contrast appears to be only due to anterior knee pain, which had unexpectedly high rates after IMN procedures among patients in a subset of studies [23,29]. Therefore, intramedullary fixation could also be associated with higher rates of knee pain at one-year follow-up; all other pain measures were equal in both groups. One study showed that when functional outcomes were assessed, the differences usually disappeared by one year [24]. This equivalence in long-term outcomes emphasizes procedures that offer faster time to recovery, such as IMN, in our study. Another study observed that after one and a half years, there were no differences in knee motion, ankle motion, fracture site pain, or ankle pain [29]. It is still the case that functional outcomes, such as joint mobility, weight-bearing, rate of chronic pain, patient satisfaction, and quality of life, should be studied more rigorously [21].

### Limitations

The limitations of this study are those familiar to single-center observational studies. It might not capture the variability seen in healthcare settings or populations, limiting external validity. The study relied on subjective measures, such as pain assessments, which can be influenced by individual patient perceptions. Variations in surgical techniques, anesthesia protocols, or postoperative care among health professionals and over the study period could introduce additional confounding. When interpreting the more frequent presence of general anesthesia in the IMN group, it should be noticed that the choice of anesthesia is, however, both anesthesiologist- and patient-dependent. In addition, as the choice of surgical technique in this study was surgeon-dependent, it should be noticed that possible selection bias might be present. However, although patients were not randomly assigned, groups were well balanced to the presence of open and closed fractures and other preoperative characteristics. As the study presents the results of early use of IMN in Serbia, the presence of a learning curve during the study period might have an effect on the duration of surgery and on the usage of antibiotics, particularly in more recent periods not presented in this study.

## 5. Conclusions

This study did not demonstrate a significant difference between postoperative complication rates and knee and ankle pain between intramedullary and external fixation. However, the two study groups differed in time to recovery, length of hospitalization, and pain intensity at the fracture site in favor of intramedullary fixation. Shorter surgery time and less antibiotic use were observed when external fixation was used. Additionally, we identified that intramedullary fixation was a significant predictor of pain intensity, and intramedullary fixation use predicted faster recovery. The length of surgery predicted the occurrence of any complication. Both methods should be compared more rigorously in multicentric randomized control trials.

## Figures and Tables

**Table 1:** Characteristics of the cohort.

Variable	Intervention		*p*
	Intramedullary Nail Fixation (*n* = 58)	External Fixation (*n* = 74)	
Sex, *n* (%)			
Male	36 (62.1)	47 (63.5)	0.865
Female	22 (37.9)	27 (36.5)	
Age, x ± sd	43.28 ± 17.12	48.31 ± 15.58	0.082
Comorbidities			
Diabetes	4 (6.9)	9 (12.2)	0.314
Hypertension	11 (19.0)	21 (28.4)	0.210
Coronary Disease	1 (1.7)	2 (2.7)	0.708
Median Length of Hospitalization in Days (IQR)	20 (15–25)	23 (19–30)	0.007
Median Surgery Waiting Times in Days (IQR)	7 (3–8)	6 (3–8)	0.912
Mechanism of Injury, *n* (%)			
Fall onto a Flat Surface	33 (56.9)	50 (67.6)	0.386
Fall from Height	3 (5.2)	6 (8.1)	
Direct Trauma	9 (15.5)	8 (10.8)	
Motor Vehicle Accident	13 (22.4)	10 (13.5)	

**Table 2:** Type of fractures in total, as well as according to type of intervention.

Type of Fracture, *n* (%)	Intervention		Type of Fracture, *n* (%)	Intervention	
	IMN (*n* = 58)	EF (*n* = 74)		IMN (*n* = 58)	EF (*n* = 74)
42-A1	10 (17.2)	33 (44.6)	42-A 94 (71.2)	42 (72.4)	52 (70.3)
42-A2	26 (44.8)	12 (16.2)			
42-A3	6 (10.3)	7 (9.4)			
42-B1	1 (1.7)	2 (2.7)	42-B 18 (13.6)	11 (19.0)	7 (9.5)
42-B2	7 (12.1)	3 (4.0)			
42-B3	3 (5.1)	2 (2.7)			
42-C1	1 (1.7)	7 (9.4)	42-C 20 (15.2)	5 (8.6)	15 (20.3)
42-C2	3 (5.1)	3 (4.0)			
42-C3	1 (1.7)	5 (6.7)			

**Table 3:** Characteristics of surgery in relation to the type of intervention for tibial fracture fixation in the study population.

Variable	Intervention		*p*
	Intramedullary Nail Fixation (*n* = 58)	External Fixation (*n* = 74)	
Anesthesia type, *n* (%)			
Spinal	26 (47.3)	56 (75.7)	<0.001
Block	8 (14.5)	12 (16.2)	
General	21 (38.2)	6 (8.1)	
Median Surgery Length in Minutes (IQR)	93 (75–130)	60 (50–80)	<0.001
Blood Transfusion, *n* (%)	1 (1.7)	0 (0.0)	0.257
Antibiotics, *n* (%)	38 (65.5)	18 (24.3)	<0.001
Low-Molecular-Weight Heparin (LMWH), *n* (%)	57 (98.3)	72 (97.3)	0.708

**Table 4:** Univariate robust regression model coefficients with recovery time treated as the outcome.

Variable	β Coefficient	*p*-Value
Sex (M/F)	0.016	0.955
Age (years)	0.014	0.063
Wait Time (min)	0.012	0.609
Intervention (nail vs. external)	−1.084	<0.001
Anesthesia		
Spinal (reference)		
Block	0.021	0.945
General	−0.095	0.792
Length of Surgery (min)	−0.001	0.889
Fracture Type		
42-A (reference)		
42-B	0.152	0.658
42-C	0.336	0.407
Mechanism of Injury		
Fall from a Flat Surface (reference)		
Fall from Height	0.650	0.297
Direct Trauma	−0.118	0.681
Motor Vehicle Accident	−0.019	0.961

**Table 5:** Univariate robust regression model coefficients with surgical complications as the outcome.

Variable	β Coefficient	*p*-Value	OR	95% Confidence Interval	
				Lower	Upper
Sex (M/F)	−0.466	0.532	0.63	0.11	2.57
Age (years)	0.023	0.273	1.02	0.98	1.07
Wait Time (min)	0.013	0.780	1.01	0.83	1.08
Intervention (nail vs. external)	0.257	0.710	1.29	0.32	5.23
Anesthesia					
Spinal (reference)		0.924			
Block	0.081		1.08	0.11	5.89
General	0.323		1.38	0.24	6.15
Length of Surgery (min)	0.011	0.015	1.011	1.002	1.021
Fracture Type					
42-A (reference)		0.204			
42-B	−0.821		0.44	0.003	4.16
42-C	1.180		3.26	0.70	13.47
Mechanism of Injury					
Fall from a Flat Surface (reference)					
Fall from Height	−0.073	0.330	0.93	0.007	9.86
Direct Trauma	1.450		4.26	0.87	19.49
Motor Vehicle Accident	0.164		1.18	0.11	6.81

## Data Availability

The data that support the findings of this study are available on request from the corresponding author.
